# The implementation and validation of the NoMAD during a complex primary care intervention

**DOI:** 10.1186/s12874-022-01655-0

**Published:** 2022-06-19

**Authors:** Larkin Lamarche, Rebecca E. Clark, Fiona Parascandalo, Dee Mangin

**Affiliations:** Department of Family Medicine, McMaster University, David Braley Health Sciences Centre, 100 Main Street West, 5th Floor, Hamilton, ON L8P 1H6 Canada

**Keywords:** Normalization process theory, NoMAD, Implementation, Complex intervention

## Abstract

**Background:**

Normalization process theory (NPT) has been widely used to better understand how new interventions are implemented and embedded. The NoMAD (Normalization Measurement Development questionnaire) is a 23-item NPT instrument based on NPT. As the NoMAD is a relatively new instrument, the objectives of this paper are: to describe the experience of implementing the NoMAD, to describe it being used as a feedback mechanism to gain insight into the normalization process of a complex health intervention, and to further explore the psychometric properties of the instrument.

**Methods:**

Health TAPESTRY was implemented in six Family Health Teams (total of seven sites) across Ontario. Healthcare team members at each site were invited to complete the NoMAD, and three general questions about normalization, six times over a 12-month period. Each site was then provided a visual traffic light summary (TLS) reflecting the implementation of the Health TAPESTRY. The internal consistency of each sub-scale and validity of the NoMAD were assessed. Learnings from the implementation of the NoMAD and subsequent feedback mechanism (TLS) are reported descriptively.

**Results:**

In total, 56 diverse health care team members from six implementation sites completed the NoMAD. Each used it at least once during the 12-month study period. The implementation of the NoMAD and TLS was time consuming to do with multiple collection (and feedback) points. Most (60%) internal consistency values of the four subscales (pooled across site) across each collection point were satisfactory. All correlations were positive, and most (86%) were statistically significant among NoMAD subscales. All but one correlation between the NoMAD subscales and the general questions were positive, and most (72%) were significant. Generally, scores on the subscales were higher at 12-month than baseline, albeit did not follow a linear pattern of change across implementation. Generally, scores were higher for experienced sites compared to first-time implementors.

**Conclusion:**

Our experience would suggest fewer collection points; three timepoints spaced out by several months are adequate, if repeated administration of the NoMAD is used for feedback loops. We provide additional evidence of the psychometric properties of the NoMAD.

**Trial Registration:**

Registered at ClinicalTrials.gov: NCT03397836.

## Background

Within health care research, Normalization Process Theory (NPT) has been widely used to understand complex interventions [1, 2]. NPT is a sociological framework that guides users to understand the implementing, embedding and integrating of complex interventions [3, 4]. NPT is composed of four constructs or mechanisms, which encompass the actions, both individually and collectively, people engage in to implement a new practice [3, 4]. First, coherence (i.e., sense-making) refers to individual and collective actions and thought processes that people do in order to operationalize something (i.e., a new intervention). Cognitive participation (i.e., relational work) involves the work individuals do to create and sustain the new practices. Collective action (i.e., operational work) is the mental and material work done to enact a set of practices. Last, reflexive monitoring involves appraisal actions where individuals aim to understand the impacts of the new practices on themselves and others. In response to the growing popularity of NPT, a toolkit was designed to facilitate critical thinking about implementation issues [5]. NPT is very versatile in its application in research and has been used prospectively to assist with the design of data collection tools and protocols, in data analyses, and to guide implementation [1, 5]. Despite NPTs flexibility, a large majority of the studies that have used NPT are qualitative, not quantitative [1, 2].

Recently, there has been an emphasis within implementation literature to quantitatively measure implementation. Common strategies to better understand implementation include assessing an organization’s readiness [6, 7], and measuring the implementation climate [8, 9], or use of an implementation framework such as the RE-AIM framework [10], which suggests using a combination of qualitative and quantitative data to gain a comprehensive look at implementation. Regarding NPT, while the NPT toolkit is useful in many contexts, it is not a validated measurement instrument thus limiting its applicability in research [5]. Therefore, the Normalization Measurement Development questionnaire (NoMAD) was developed; a pragmatic, psychometrically tested 23-item instrument which measures implementation activities [11–13]. The NoMAD has been used to assess the implementation of complex interventions within diverse contexts including a physician-based weight management program [14], and a surgical checklist among health care professionals [15].

Although the questionnaire has demonstrated face and construct validity [5] and has been translated and adapted cross-culturally [16–18], evidence for the NoMAD’s psychometric properties is in its infancy. For example, information about its criterion-related validity and test–retest reliability is sparse [12]. Further there is minimal information about administration of the NoMAD in primary care settings. Finch et al. [12] emphasized the pragmatic nature of the NoMAD, consistent with the need for ‘pragmatic measures’ that balance sound psychometric properties with usability in a real-life context. Therefore, the objective of this paper is to describe the experience using the NoMAD as a novel feedback tool within a complex primary care intervention, and to assess and provide data on some psychometric properties of the NoMAD.

## Methods

### The complex intervention

Health TAPESTRY (Health Teams Advancing Patient Experience: STRengthening qualitY) is composed of four parts: an interprofessional primary health care team, use of technology to facilitate the collection and sharing of client (patients enrolled in the program) information, trained volunteers to collect information from clients and the facilitation of community engagement and connections [19, 20]. As a whole, Health TAPESTRY aims to enable more coordinated, comprehensive and person-centred care for patients, which are key contributing factors to a strong primary care [21]. Strengthening primary care is an effective way to improve patient and service use outcomes [22–24].

Operationally, clients in the program are visited by two community volunteers who ask a comprehensive series of surveys that address the clients’ health needs and personal goals. The information is entered into a tablet application, summarized into a report, which is then received by a small group of interprofessional health care providers (IHPs) (e.g., pharmacist, dietician, physician) linked to the client’s own primary care practice. The group, termed the TAP-Huddle, jointly creates a plan of action based on the report and facilitates the plan. Plans can have a variety of tasks including referrals, follow-up appointments with a physician, or the request for patient friendly resources to be mailed. Data for this study come from the larger trial assessing the feasibility and reproducibility of the results of Health TAPESTRY (see [16] for full protocol).

### Study implementation sites and participants

Health TAPESTRY was implemented in Family Health Teams (FHTs) in six communities across Ontario, Canada. FHTs are primary care organizations that formally links physicians and a variety of health care professionals such as nurses, dietitians, and occupational therapists [25]. The interprofessional team members may be co-located or at different locations, depending on the local FHT context. One FHTs had two sites resulting in two TAP-Huddles. Eligible study participants were primary care staff involved with the program either as a TAP-Huddle member (e.g., dietitian) or involved in the management of the program (e.g., administration). Leaders within the primary care practices helped the research team determine the appropriate individuals to invite to participate throughout the duration of the study as the membership of the huddles changed over time.

### Data collection

**Fig. 1:**
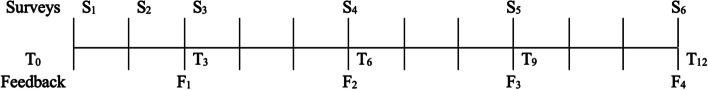
Study and feedback timeline over one year

The IHPs were invited to complete the survey through an electronic survey link (i.e., LimeSurvey [26]). The research team invited participants to complete the NoMAD six times over the course of one year during implementation (Fig. 1, [5]). The authors of the NoMAD encourage users to apply any necessary modifications to the instrument to improve its relevance to the study’s objectives [5]. The word intervention in each question was replaced with ‘Health TAPESTRY’ and one question was re-worded in the reflexive monitoring subscale. The original question read; “I am aware of the reports about the effects of [the intervention]” and the question was re-worded to; “I am aware of reports about the effects of Health TAPESTRY". The surveys were voluntary, and participants could withdraw at any point. Informed consent was obtained for all participants. Data was collected from April 2018 to January 2020. This study received ethics clearance from the Hamilton Integrated Research Ethics Board (#3967) and all methods were performed in accordance with relevant guidelines and regulations.

### Scoring and feedback summaries

The NoMAD is a 23-item questionnaire; the first three questions are general questions about the intervention, and the remaining 20 questions are more detailed. The NoMAD has two categories of answers for each of the 20 detailed question; Option A had five response options (strongly disagree to strongly agree) and Option B, which participants used if the question was irrelevant to their role, the intervention or to the stage in implementation. While the NoMAD does not have a structured scoring template, we applied a five-point Likert scale to all responses (1 = strongly disagree, 3 = neither agree nor disagree, 5 = strongly agree) used by Gillespie et al. [15]. One item within the collective action subscale was reverse coded. The three general questions at the start of the NoMAD (rated on a scale of 0–10, with higher scores more favourable to normalization) address how familiar the intervention feels (called Familiar), if the intervention feels part of the respondents work currently (called Current), and if the respondent feels the intervention will become a normal part of work (called Future).

**Fig. 2:**
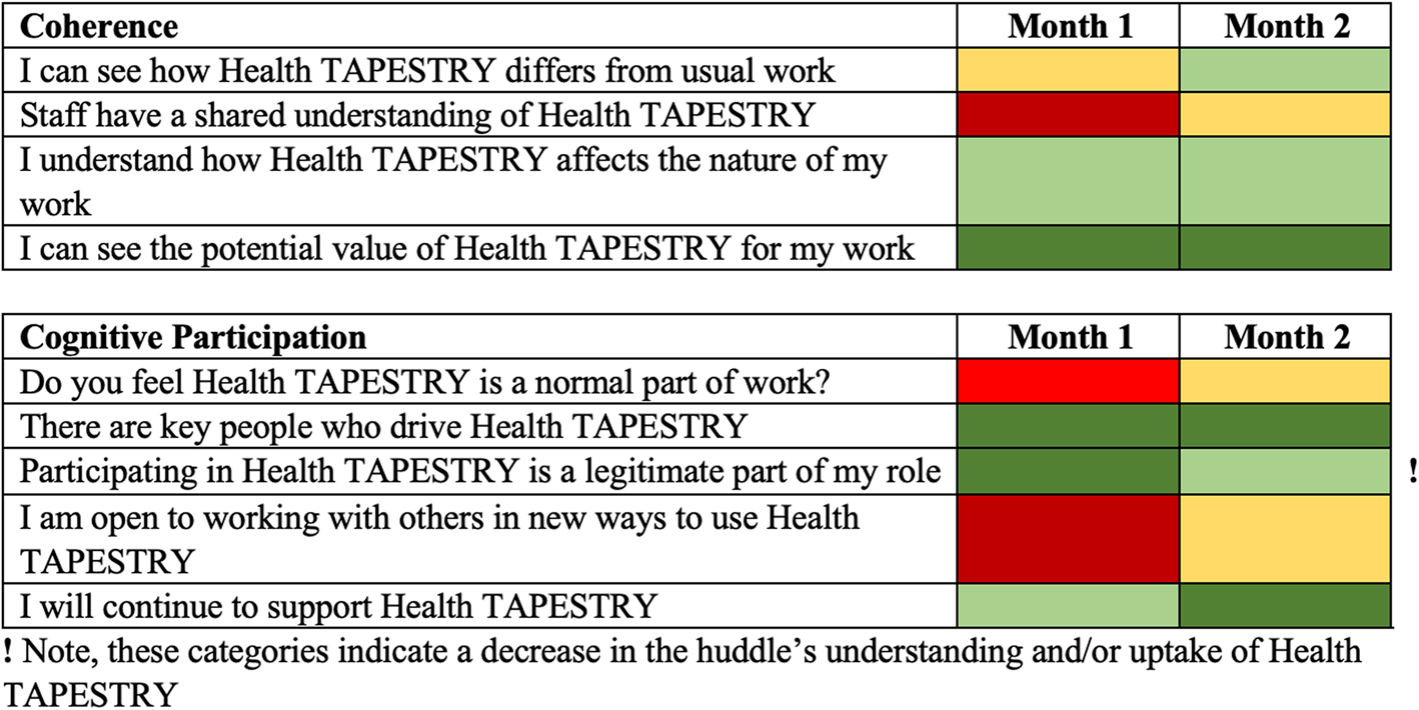
A sample TLS for sensemaking and engagement, two months after implementation

To provide the TAP-Huddles feedback regarding the implementation of Health TAPESTRY, we sent aggregate participant data for the site four times over one year to each site’s TAP-Huddle lead, and anyone else at the site who requested it (Fig. 1). The feedback was presented resembling a resembling a traffic light, a method based on Reeve et al. [27]. The traffic light summary (TLS) provided a visual representation of how the TAP-Huddle is understanding and normalizing the program at that time point. The colour reflects the mean (1.0–1.5 = dark red, 1.6–2.5 = red, 2.6–3.5 = yellow, 3.6–4.5 = light green, 4.5–5.0 = dark green). The feedback contained data for each question, and the NPT constructs. A written narrative of the values accompanied the TLS when it was sent to a team. If a team was moving in a negative direction (i.e., Health TAPESTRY was becoming less normalized) compared to the previous time point, that was indicated with both the colour coded visualisation and an extra flag that was indicated with both the colour coded visualisation, extra flag and a written statement in email correspondence (Fig. 2). No further instructions were provided to the TAP-Huddle leaders. Email correspondence and meeting notes were used to gather each TAP-Huddle’s impression of, and feedback on, the NoMAD and TLS as well as document any challenges to implementing the NoMAD.

### Data analysis

For insight into the usefulness and key learnings regarding the use of the NoMAD and TLS, we completed an audit of the research team’s emails, meeting minutes and feedback from key individuals involved in the implementation and evaluation of Health TAPESTRY. We conducted several psychometric assessments. We calculated Cronbach’s alpha (α) for each subscale by time point (pooled by site) to assess internal consistency. A value of 0.70 or higher was deemed satisfactory; a score of 0.90 was deemed high [20]. To explore possible floor and ceiling effects of the NoMAD, we calculated the range of scores at each time point and the skewness of each subscale, pooled across site. Skewness values between + 1 and -1 were identified as satisfactory. To assess construct validity, bivariate correlations were calculated between subscales and between subscales and the general questions (Familiar, Current, Future) by time point, pooled across site. Correlations were classified as strong (± 0.70), moderate (± 0.40 to ± 0.60), and weak (± 0.30 to ± 0.10) [21]. To assess concurrent validity, hypothesized NoMAD differences were explored between baseline versus 12-month time points pooled by site (and separately by site). We also examined hypothesized differences between first-time implementers versus second-time implementers at baseline. Given our expected small sample size, no planned statistical analyses were completed to test this hypothesis, rather patterns of difference (using means and standard deviations) were visually inspected and described to inform future work. All analyses were done with IBM SPSS (Version 26) software.

## Results

A total of 56 IHPs completed the NoMAD at least once throughout the study, 34% of respondents completed all surveys, and a further 18% completed at least three of the NoMAD surveys. Almost half of the respondents were allied health professionals, and most were TAP-Huddle members (Table 1). Respondents per site per timepoint ranged from 2–8 (Table 2).

**Table 1:** Participant demographics (*n* = 56)

Position	N (%)
Management	6 (10.71)
Administrative staff	4 (7.14)
Physician	6 (10.71)
Nurse	12 (21.43)
Allied health	25 (46.43)
Held multiple positions	2 (3.57)

**Table 2:** Number of respondents per site per timepoint

	**Site 1**	**Site 2**	**Site 3**	**Site 4**	**Site 5**	**Site 6**	**Site 7**
Month 1	7	3	7	4	5	5	5
Month 2	7	2	8	4	3	5	3
Month 2	4	2	6	5	4	4	2
Month 6	5	2	3	3	3	2	5
Month 9	5	2	5	2	4	3	2
Month 12	8	2	7	3	3	2	2

One site had two TAP-Huddles which is why there are 7 sites in the table

### The administration of the NoMAD and TLS

Overall, we found it was time consuming to recruit, retain, and remind participants throughout the study to complete the NoMAD multiple times at the implementation sites. Further, the TAP-Huddle composition at each site changed over time, often as a result of clinical role and workload changes. Therefore, time was spent, often relying on timely communication from the primary care site leads, by the research team at each timepoint determining the individuals who should and should not receive the NoMAD as part of the TAP-Huddle.

There were a few key learnings regarding the implementation of the TLS. The original TLS based on Reeve et al. [27] only had three colours (red, amber and green). Early in implementation, two other colours (dark red and light green) were added in response to suspected ceiling effects of the NoMAD when sites were reporting high scores (indicating more normalization) very early in implementation. More colours allowed participants to see more subtle changes in their normalization of Health TAPESTRY, especially if they scored ‘green’ early on. We are unsure of how consistently the TLS were viewed by sites, or if the information was presented to all the TAP-Huddle members as it was typically only sent to the TAP-Huddle lead at each site. We know at least two TAP-Huddle leads viewed the TLS, but we felt no further actions were needed to improve the implementation and embedding of Health TAPESTRY. Throughout the study, preparing the TLS was time consuming for the research team.

### Psychometric assessment

Most values for Cronbach alphas (*n* = 20, 60%) for all four of sub-scales (pooled across site) by time point reached satisfactory thresholds (i.e., α ≥ 0.70). One value (for cognitive participation, month two) reached a high threshold level (α = 0.91). Four values did not reach a satisfactory threshold (Coherence, month 3, α = 0.66; Coherence, month 9, α = 0.33; Coherence, month 12, α = 0.45; Collective Action, month 12, α = 0.67). Table 3 shows the range of means (standard deviations), range of skewness values, and range of correlations across time (pooled by site). All means for all subscales were above the mid-point of the scale, and standard deviations values were small (below 1.0). Most (*n* = 22, 92%) skewness values were below ± 1.0, including two that hovered around ± 1.0 (-1.3 for Coherence, month 6 and 1.24 for Coherence, month 9). Two values were outside acceptable values at month 1 (Coherence, month 1, skewness, SE = -1.76, 0.40 and Cognitive Action, month 1, skewness, SE = -2.13, 0.39). Table 4 shows the range of correlations (pooled by site) among subscales of the NoMAD and between subscales of the NoMAD and the three general questions (Familiar, Current, Future). All relationships among NoMAD subscales were in the positive direction. Most (*n* = 29, 86%) of the relationships among NoMAD subscales were statistically significant (seven were not statistically significant, *p* > 0.05). Most (*n* = 17, 59%) statistically significant relationships were strong in strength. The remaining statistically significant relationships (*n* = 12, 41%) were moderate in strength. Most (*n* = 52, 72%) of the relationships between NoMAD subscales and the three general questions were statistically significant. All but one relationship (between Coherence and Future, month 1, *r* = -0.01, *p* = 0.94) was (negligibly) in the positive direction. Most (*n* = 44, 85%) of the significant relationships were moderate in strength, with the remaining eight (15%) strong in strength. Table 5 shows baseline and 12-month NoMAD subscale scores (pooled by site). Coherence, Collective Action, and Reflexive Monitoring were (descriptively) higher at 12-months versus baseline, and Cognitive Participation was lower, albeit negligibly. Visual inspection of scores of the NoMAD across time within each site indicated that the normalization of Health TAPESTRY was not a linear increase from baseline to 12-months (data not shown). All sites, at least once, regressed on one or more sub-scales throughout the 12-month implementation period. Descriptively, the two sites that previously implemented Health TAPESTRY (i.e., experienced sites) had higher baseline values for three of the four subscales than first-time implementors: coherence, mean(SD) = 4.06(0.57) versus 3.89(0.85), cognitive participation = 4.46(0.37) versus 4.39(0.55), and collective action = 4.00(0.40) versus 3.94(0.69), respectively. The difference of means between the second-time implementors compared to the first-time for reflexive monitoring was small: 3.85(0.49) versus 3.89(0.60).

**Table 3:** Range of means (SDs), skewness (SEs) values, correlations for NoMAD subscales across implementation

Subscale	Mean (SD) range	Skewness (SE)	Cognitive Participation	Collective Action	Reflexive Monitoring
Coherence	3.95 (.76) - 4.15 (.40)	-1.76 (.40)—1.24 (.48)	0.29—0.72	0.13—0.74	0.29—0.71
Cognitive Participation	4.25 (.50) - 4.44 (.47)	-0.38 (.40)—0.118 (.50)	–-	0.39—0.78	0.49—0.79
Collective Action	3.94 (.69) - 4.10 (.44)	-2.13 (.39) - 0.49 (.45)		–-	0.60—0.83
Reflexive Monitoring	3.88 (.56) - 4.07 (.56)	-0.19 (.45) - 0.20 (.41)			–-

Scores range from 1 to 5, higher values represent higher normalization for that subscale; Scores pooled across site; All correlations were positive values

*SD* Standard Deviation, *SE* Standard Error

**Table 4:** Range of correlations for sub-scales and overarching questions across implementation

Subscale	Familiar	Current	Future
Coherence	0.13—0.50	0.20—0.62	-0.01^a^—0.71
Cognitive Participation	0.19—0.61	0.23—0.62	0.20—0.64
Collective Action	0.47—0.82	0.25—0.71	0.21—0.69
Reflexive Monitoring	0.30—0.60	0.27—0.62	0.33—0.56

^a^ All correlations except for one value were positive

Sub-scale scores range from 1 to 5, higher values represent higher normalization; Scores pooled across sites; General questions (Familiar, Current, Future) scores range from 0 to 10, higher values represent higher normalization for that subscale; Familiar: how familiar does it feel, Current: does it feel like a normal part of work, Future: feel it will become normal part of work

**Table 5:** Baseline and 12-month NoMAD subscale scores

Subscale	Baseline	12-month
Coherence, mean (SD)	3.95 (.76)	4.01 (.46)
Cognitive Participation, mean (SD)	4.35 (.50)	4.32 (.51)
Collective Action, mean (SD)	3.94 (.69)	4.10 (.42)
Reflexive Monitoring, mean (SD)	3.88 (.56)	4.06 (.47)

Scores range from 1 to 5, higher values represent higher normalization for that subscale; Scores pooled across sites

*SD* Standard Deviation

## Discussion

Our study described our experience using the NoMAD as a novel feedback tool within a complex primary care intervention and assessed some psychometric properties of the NoMAD. We identified some key learnings using the TLS and the NoMAD. First, we sent the IHPs the NoMAD six times, which when considering the time to administer and facilitate the questionnaires to multiple sites at different time points, and occasionally low response rates, we believe would be too much for both sites and researchers or program implementers to use routinely. Instead, we suggest implementing the NoMAD only three times; early, mid- and late implementation. We believe this can provide an opportunity to see how well an intervention is being normalized and respond, while not burdening the participants. Second, we generated the TLS using means from the NoMAD. We wondered whether the instinct of healthcare teams to identify problems/challenges/gaps and find solutions underscored the important role the TLS may play in feedback to teams about ‘what is going right.’ Even though we adapted the TLS to share feedback about subtle changes in normalization, the tight range of values and high scores we had in our data make us believe another type of measure may be more appropriate to use in the future of Health TAPESTRY. Third, we caution users that plan to solely rely on the NoMAD as an indictor of normalization. While many of the findings from the NoMAD were congruent with the results of the robust qualitative analysis of implementation as part of the larger trial (described elsewhere), the qualitative data provided additional information about implementation that is arguably of equal importance. For example, physician buy-in (i.e., physicians not directly involved in the implementation of Health TAPESTRY was as an issue for primary care sites, which would not be captured by the NoMAD as it is completed by and asks questions relating only to the staff involved with implementing Health TAPESTRY.

In general, our results regarding the NoMAD’s psychometric properties demonstrated internal consistency and provided some evidence of validity, consistent with past work literature [12, 15] as well as add to the minimal literature based in primary care. However, we want to comment on a few useful findings. First, Coherence (month 9 and 12) had very low Cronbach’s alphas indicating that responses do not make consistent sense. Unpacking this further we notice that the first question (“I can see how Health TAPESTRY differs from usual work”), in some cases, does not align with the others in the subscale. For example, one person responded to the first question with “strongly disagree,” but with “strongly agree” to the other questions (e.g., “I understand how Health TAPESTRY affects the nature of my work”) in the subscale. This may be a function of the team members who responded to the survey across implementation (a limitation noted below); the make-up changed such that people responding in month 12 may have been new to the TAP-Huddle. However, this flux in sample make-up did not seem to impact other subscales across implementation; highlighting the balance between psychometric soundness and useability in real-life that pragmatic measures like the NoMAD accomplish, a point emphasized by Finch et al. [12]. In terms of validity, with a few exceptions, we provided additional evidence of validity for the NoMAD, with the findings of positive relationships among subscales of the NoMAD (as expected), positive relationships between the subscales and the three general questions (as expected), and expected descriptive differences between first-time and experienced sites at baseline and between baseline and late implementation timepoints.

Interestingly, when tracking scores across implementation, we noted a non-linear trajectory. This too may be a function of different people that comprise the samples at each time point in implementation, and perhaps from a wider pragmatic perspective, reflects *how* normalization may occur across implementation as something of a process and not an end point. From the qualitative analysis, we know the primary care sites had changes in workflow as a result of the program such as more frequent interdisciplinary team work which, would certainly impact the normalization process. Further, Health TAPESTRY as a complex intervention had several moving parts, and served as an ongoing PDSA (Plan, Study, Do, Act; [22] cycle whereby feedback from stakeholders (e.g., a volunteer) may have led to adjustments in the process, providing an opportunity to learn from the adjustment. This continuous state of learning can be a challenge to achieve an endpoint of absolute “normalization” but does reflect the real-world context of implementation in the primary care setting.

### Strengths and limitations

A limitation of this study is we did not qualitatively gather feedback from TAP-Huddles regarding the TLS, but instead, relied on secondary sources of information. Second, we could not conduct statistical tests to assess validity because our sample was small and, for the examination of differences between baseline and 12-months, and was not paired (i.e., the same team members did not complete the surveys each time: the NoMAD was administered within a larger pragmatic trial, therefore the TAP-Huddle members changed over time, therefore the respondents changed). Further, given the target population (i.e., busy IHPs) and repeated measures, we did not have a 100% response rate from each site at every time point. Having the same people complete the NoMAD over the course of implementation would allow for a more robust assessment of some types of psychometric properties (i.e., sensitivity to change over time, test–retest reliability). This is also a strength in assessment of the utility of NoMAD as it reflects the changing real world of implementation contexts (i.e., staff turnover), and illuminating the pragmatism of NoMAD [12]. A further strength of the study was the tracking of the NoMAD over one year of implementation, allowing us to get a comprehensive look at normalization over time, and investigate the usability of the NoMAD in this way for a complex, multi-site randomized controlled trial in primary care.

## Conclusion

Based on our experience with the TLS of the NoMAD as a feedback tool within a complex, multi-site trial we make some suggestions for most effective use. Too many feedback points, too close together in time makes for cumbersome collection, and potentially meaningless, feedback loops. Careful thought and planning are needed about when it makes the most sense to provide feedback to implementation sites. This will depend on the time frame for implementation, the nature of the intervention and the time needed to make adjustments relating to implementation. For a 12-month trial we suggest three collection points (early, middle, and late implementation) are adequate, with a few months between collection points. Due to the nature of pragmatic measures such as the NoMAD, we suggest scholars report basic psychometric properties (e.g., internal consistency) in their published work as ongoing evidence to support use and improvement of implementation of the NoMAD as well as performance in different contexts.

## Data Availability

The datasets used and/or analyzed during the current study are available from the corresponding author upon reasonable request.

## Abbreviations

FHT: Family Health Team
Health TAPESTRY: Health Teams Advancing Patient Experience: STRengthening quality
IHP: Interprofessional health care providers
NoMAD: Normalization Measurement Development questionnaire
NPT: Normalization Process Theory
TLS: Traffic Light Summary
